# Microbial Distributions Across Wide‐Ranging Temperature Gradients of Hot Springs in Thailand: A Review of 35 Years of Research on Hot Spring‐Associated Microorganisms

**DOI:** 10.1111/1758-2229.70302

**Published:** 2026-02-18

**Authors:** C. Sriaporn, K. Phinyo, U. Sompong, J. Pekkoh, J. Homnan, S. Komonjinda

**Affiliations:** ^1^ Research Group on Space Weather and Cosmic Rays From Ground‐Based Observations and Effects on Earth‐Space Ecology Chiang Mai University Chiang Mai Thailand; ^2^ Algal and Cyanobacterial Research Laboratory, Department of Biology, Faculty of Science Chiang Mai University Chiang Mai Thailand; ^3^ Office of Research Administration Chiang Mai University Chiang Mai Thailand; ^4^ Faculty of Fisheries Technology and Aquatic Resources Maejo University Chiang Mai Thailand; ^5^ Department of Lithospheric Research, Faculty of Earth Sciences, Geography and Astronomy University of Vienna Vienna Austria; ^6^ Department of Physics and Materials Science, Faculty of Science Chiang Mai University Chiang Mai Thailand

**Keywords:** ecological distribution, hot spring, microbial communities, temperature, Thailand

## Abstract

Although Thailand harbours > 100 hot springs with distinct physicochemistry for diverse microbial communities, an extensive biogeography of hot spring microbial communities has not previously been explored. Here we summarised the progression of previous research and their findings on microbial distributions using data gathered from 62 hot springs (temperature = 32°C–90°C, pH = 6.2–10.0), retrieved from 51 documents (from 1990 to 2025), spanning across 21 provinces along the northern, central, eastern, western and southern regions of Thailand. Cyanobacteriota communities were the most characterised, with *Synechococcus* and *Phormidium* as frequently reported genera among studies. Several cyanobacterial members, for example, *Pseudanabaena*, *Synechococcus*, *Chroococcus* and *Chroococcidiopsis* were reported at remarkably wide temperature ranges. For non‐photosynthetic bacteria, Pseudomonadota and Bacteroidota were the majority of the communities, while members from Bacillota were the most isolated. Pseudomonadota also harbour the widest distribution range, spanning ~70°C across sites. However, temperature measurements from some studies might not accurately reflect in situ conditions. Additionally, archaea were rarely noted in the community likely due to methodological limitations. Microbial eukaryotes, for example, green algae and diatoms, were occasionally isolated and classified but their communities remain largely uncharacterised. This review provides the first integrated overview and reference database of hot spring‐associated microorganisms in Thailand.

## Graphical abstract

This review article summarised 35 years of research on microbial distributions (bacteria, archaea and microbial eukaryotes) across wide‐ranging hot spring temperatures in Thailand, including the overall research progression, scopes and methodologies of previous studies.

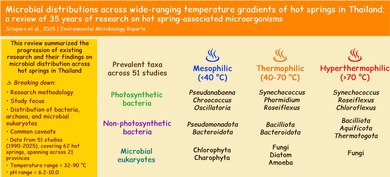

## 1 Introduction

Phylogenetically diverse microorganisms are found across wide‐ranging physicochemistry of terrestrial hot springs globally, such as Yellowstone National Park (YNP, USA) (Hugenholtz et al. 1998), Taupō Volcanic Zone (TVZ, New Zealand) (Power et al. 2018), Hveragerði (Iceland) (Krebs et al. 2014), Japan (Nishiyama et al. 2018), El Tatio (Chile) (Sanchez‐Garcia et al. 2019), Kamchatka (Russia) (Wemheuer et al. 2013) and including Thailand (Pointing et al. 2024). Extremophilic microorganisms are generally adapted to the extreme temperatures and pH values of hot springs, but in some cases their growth can also occur under milder conditions such as moderate temperatures or near‐neutral pH (Brock et al. 1972; Stetter 2006). In general, hot spring microbial community composition changes with shifts in physicochemistry such as temperature, pH and water chemistry (Sharp et al. 2014; Power et al. 2018; Sriaporn et al. 2020). High‐temperature hot spring environments (> 70°C) are normally occupied by thermophiles, such as the archaeal phylum Thermoproteota (Barns et al. 1994) and the bacterial phylum Aquificota (Reysenbach et al. 2000), whereas, in moderate temperature hot spring environments (40°C–70°C), mesothermophilic bacterial phyla such as Pseudomonadota, Thermotogota and Epsilonbacteraeota, including the archaea Euryarchaeota, become the primary prokaryotic groups (Meyer‐Dombard et al. 2005; Power et al. 2018). On the other hand, low‐temperature hot spring environments (< 40°C) are primarily occupied by bacterial phyla such as Cyanobacteriota and Bacteroidota, with little‐to‐no archaea (Power et al. 2018). Similarly, hot spring microbial community composition can also be affected by pH. Highly acidic hot springs, which are commonly coupled with high temperatures, are populated mainly by thermoacidophiles such as Thermoproteota and Euryarchaeota (Barns et al. 1994; Meyer‐Dombard et al. 2005; Reigstad et al. 2010; Eme et al. 2013; Hou et al. 2013). In contrast, alkaline hot springs (pH > 7), where temperatures are ≤ 73°C, are generally occupied by thick, colourful photosynthetic mats of mainly Cyanobacteriota and Chloroflexota, which reside in the upper layers of microbial mats, with heterotrophic communities in the lower mat layers (Klatt et al. 2011).

Extremophiles are able to live in extreme temperatures because they possess structural and physiological adaptations that distinguish them from non‐extremophiles (Adams 1993). By increasing the amount of saturated fatty acids, long‐chain fatty acids and branched‐chain fatty acids (Ray et al. 1971; Sinensky 1971; Oshima and Miyagawa 1974), bacteria are able to live in high‐temperature environments. Bacteria may also possess heat shock proteins, which help correct misfolded proteins affected by high heat and activate repair mechanisms (Yura et al. 1993). In archaea, heat‐tolerant proteins help increase the ability to retain structures and functions in high temperatures (Reed et al. 2013). Furthermore, archaea also have tetraether linkages in their cytoplasmic membranes that allow them to maintain membrane integrity in high‐temperature environments (Woese et al. 1978; Albers et al. 2000). Bacterial adaptations to highly acidic environments involve mechanisms that pump out excess intracellular protons and bring in other positively charged solutes in order to maintain the charge balance, or dispense protons via other metabolic processes, such as amino acid decarboxylation (Baker‐Austin and Dopson 2007; Lund et al. 2014). In contrast, increases in cellular proton influx, boosting intracellular acid production and retention of cytoplasmic protons help maintain pH homeostasis in alkaline settings (Padan et al. 2005).

**FIGURE 1:**
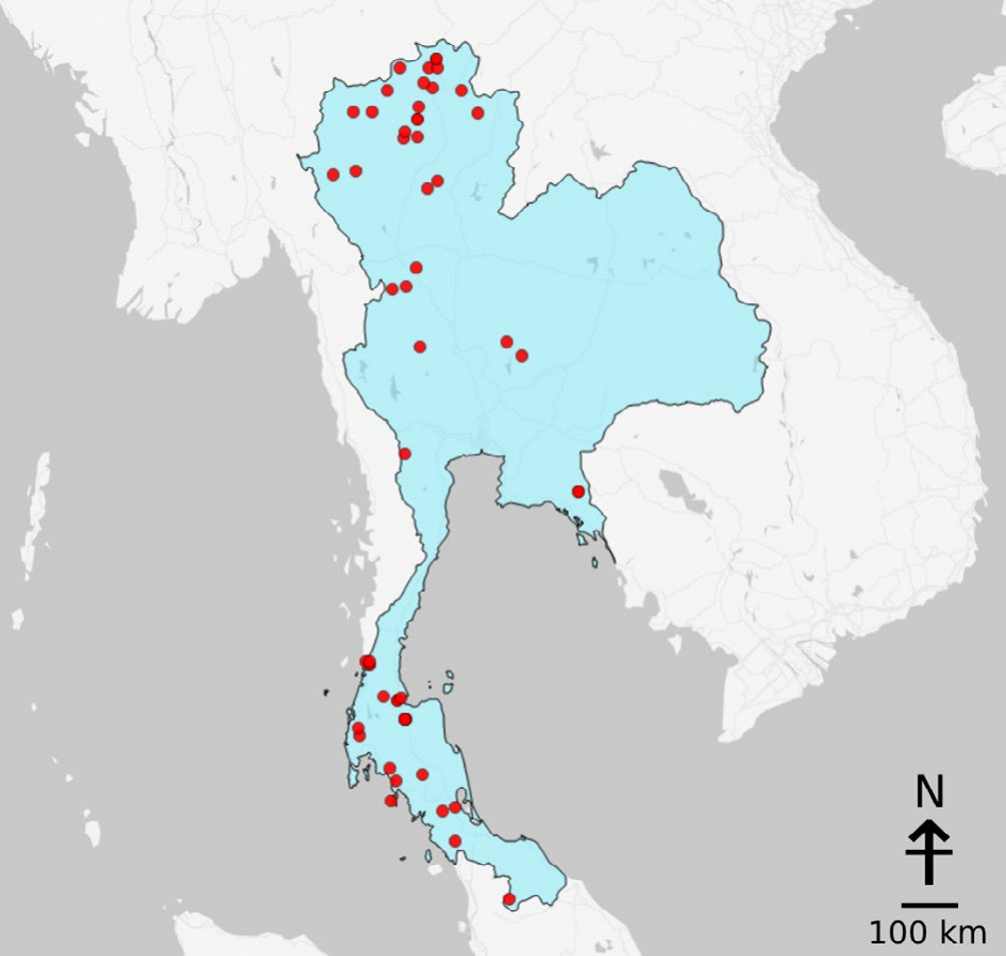
Map of the hot springs in Thailand included in this review. Fifty‐three out of 62 hot springs were marked in this map. The remaining nine hot springs were unmarked because of missing identifiers in their original studies. Note that marks for certain sampling sites may overlap because these hot spring pools are located in close proximity to one another.

In Thailand, there are over 100 terrestrial hot springs, which encompass physicochemically distinct environments and harbour phylogenetically diverse microbial lineages. However, an extensive national biogeography of hot spring microbial communities has not previously been explored. In this review, we summarised the progression of previous research and their findings on hot spring microbial distributions, using data from 51 studies conducted over the past 35 years (1990–2025), covering 62 hot spring sites across 21 provinces along the northern, central, eastern, western and southern regions of Thailand (Figure 1, Table S1). We found that studies on hot spring‐associated microorganisms in Thailand spanned a variety of research scopes and were conducted through a combination of traditional and modern techniques. Existing literature is heavily involved with bacterial lineages (> 90% overall). Shared photosynthetic taxa across Thai hot springs included Cyanobacteriota and Chloroflexota members such as *Synechococcus*, *Pseudanabaena*, *Roseiflexus* and *Chloroflexus* in the high‐temperature sites, with *Leptolyngbya*, *Calothrix*, *Oscillatoria* and *Phormidium* distributing along low‐to‐moderate‐temperature springs. Furthermore, non‐photosynthetic bacteria of hot spring communities in Thailand included Pseudomonadota, Deinococcota, Bacillota, Aquificota and Thermotogota, which were the main residents of high‐temperature settings, while moderate‐ and low‐temperature springs were occupied by Bacteroidota, Acidobacteriota, Actinomycetota and Planctomycetota. Documentation of archaea only accounted for a minor fraction, likely due to methodological limitations. Microbial eukaryotes were generally identified, but their communities remain largely uncharacterised. Since other environmental variables, such as pH and sulphide, vary minimally across hot springs in Thailand, temperature is one of the main drivers of microbial community composition, although a few members from certain phyla, such as Cyanobacteriota and Pseudomonadota, were present across wide‐ranging hot spring temperatures. Overall, this review is among the first to provide a comprehensive summary of microbial communities across hot springs in Thailand, establishing a definitive national reference and serving as a microbiological database for these unique environments.

## 2 Methods

### 2.1 Search Strategy and Screening

Combinations of keywords listed in Table S2 were used to construct search strings, which were applied to Google, Google Scholar and institutional digital repositories of Chiang Mai University, Chulalongkorn University, Mahidol University and Thammasat University, which are among the top‐ranking universities in Thailand (according to QS World University Rankings 2026; https://www.topuniversities.com/world‐university‐rankings). Search results were examined through all available pages, and studies were included in this review if they met the following criteria:

1. The study was published between 1990 and 2025 (up to October 2025, at the time of writing).
2. The study site (or where samples were collected from) must be hot spring environments or hot spring‐influenced environments in Thailand.
3. Hot spring temperature must be ≥ 32°C, following criteria established by Department of Mineral Resources (DMR 2011), Thailand.
4. The study must include analysis of microorganism/microbial component/microbial product from hot spring environments.
5. Results must indicate positive detection of microorganism/microbial component/microbial product, although taxonomic classification is not required.
6. In cases of duplicated research outputs, for example, theses later published as journal articles or multiple journal articles derived from the same study with only slight variations in context (such as genome announcements later expanded into full studies), only the most recent and extensive version was retained.
7. Publications referring to unspecified or unnamed hot springs, even when produced by the same research group, were treated as independent studies when site verification was not possible.

A total of 57 records were retrieved from multiple publication sources and screened for relevance and originality. Publications sharing substantial overlap in data or context were carefully examined to avoid redundancy, and 51 studies were retained for analysis. Details of the screening outcomes for each publication source are provided in Table 1. Nomenclatures of all taxonomic classifications, especially those from older publications, have been updated according to Oren and Garrity (2021) and Oren and Göker (2023).

**TABLE 1:** Overview of records collected from diverse publication sources considered during the screening stage.

Source type	Total records retrieved	Multiple outputs from the same study	Later converted to journal article	Final records included
Journal article	43	3	N/A	40
Doctoral thesis	1	0	1	0
Master thesis	9	0	1	8
Undergraduate dissertation	1	0	0	1
Conference proceeding	1	0	0	1
Grant report	2	0	1	1
Total	57	3	3	51

### 2.2 Statistical Calculation

Overall temperature and pH values were reported as median ± interquartile range (IQR) due to their non‐normal distribution. All percentage‐based calculations used the total number of studies (*n* = 51) as the denominator (i.e., presence of *X*/51), unless specified otherwise. The number of hot spring sites was not used as a denominator because several studies sampled multiple springs but reported combined results, with no site‐specific data.

### 2.3 Primer Coverage Evaluation

Evaluation of primer set coverages of profiling studies was calculated using the SILVA TestPrime tool (https://www.arb‐silva.de/testprime) against the SILVA database SSU‐138.2 with RefNR collection and 0 mismatches.

Primer coverage percentages were reported as mean ± standard deviation (SD), as too many studies exhibited zero coverage. The coverage mean was calculated as the sum of each primer's coverage multiplied by the number of studies that used it, divided by the total number of studies considered. Other non‐community profiling studies, for example, isolation studies, were calculated as if their coverage was zero, as these studies only identified one or a few species. The weighted standard deviation was calculated as the square root of the sum of each primer's weighted squared deviation from the weighted mean, divided by the total number of studies.

### 2.4 Visualisation

Visualisation of ternary diagram was generated using RStudio (version 4.2.2) with R package ggtern (version 3.5.0). Visualisation of other plots and charts were generated with R package ggplot2 (version 3.5.1). Map was generated using web‐based software Map Maker (https://maps.co/).

## 3 Geology and Geochemistry of Hot Springs in Thailand

**FIGURE 2:**
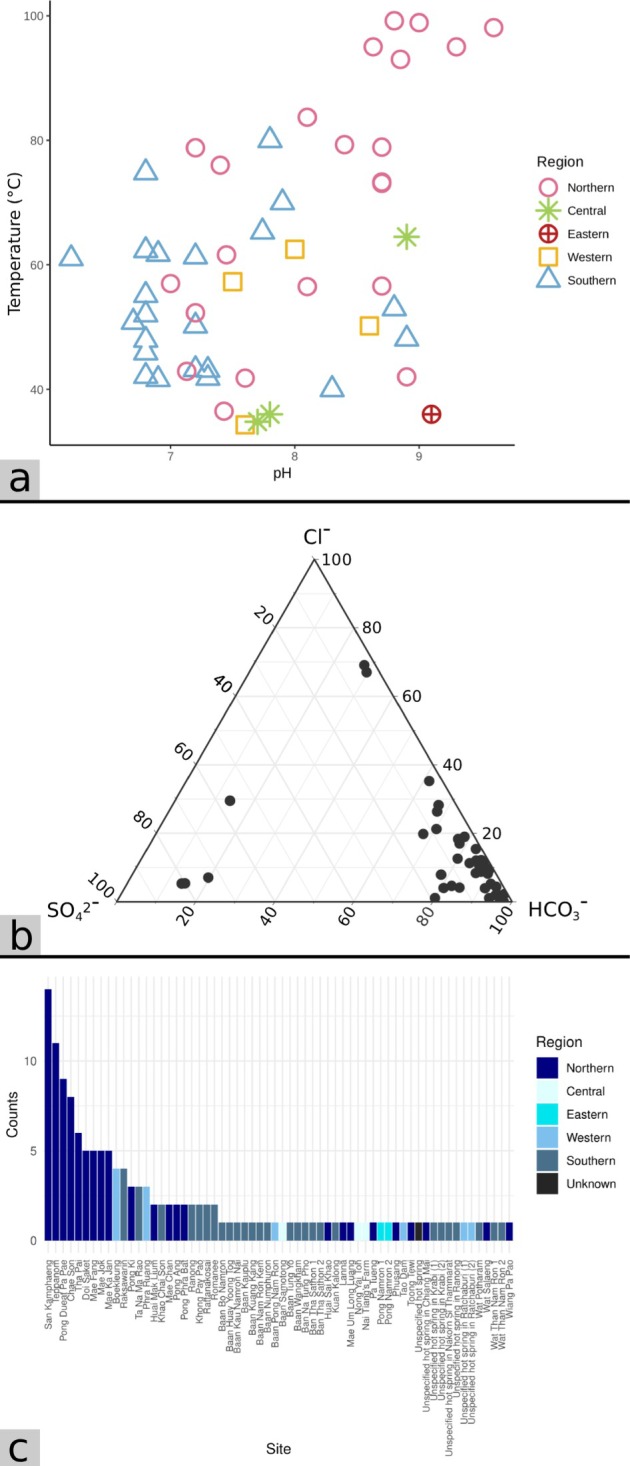
(a) Reference distribution of temperature and pH of hot springs in Thailand, grouped by region. Data represent reference temperature and pH values were compiled from Table S3 (*n* = 54). (b) Reference geochemical composition of hot spring waters in Thailand (Table S3) (*n* = 54). (c) Number of studies conducted at each Thai hot spring site. Each bar represents the total count of independent studies that reported data from a given site.

At present, the DMR of Thailand has documented at least 112 terrestrial hot springs in Thailand, which altogether are associated with > 10 major active faults (Figure S1). Hot springs in Thailand are predominantly situated on non‐volcanic terrain, where high‐angle faults and fracture networks in crystalline and sedimentary rocks control hydrothermal circulation. Among the major fault zones in northern Thailand, the Mae Chan Fault Zone, trending east‐northeast to west‐southwest (ENE‐WSW) direction across Chiang Mai and Chiang Rai provinces, is a major strike‐slip structure that has remained active since the Cenozoic and hosts several hot springs, including those in the Fang and Mae Chan areas (Singharajwarapan et al. 2012; Wood and Singharajwarapan 2014). Similarly, the Mae Tha Fault, located east of the Chiang Mai Basin, exerts strong structural control on the San Ka mphaeng, Doi Saket and Chae Son geothermal systems. These systems are developed mainly in granitic and metamorphic terrains, where reactivation of pre‐existing fracture zones during Cenozoic extension created permeable conduits that allow deeply circulated meteoric water to ascend (Wood and Singharajwarapan 2014). The Mae Hong Son Fault, located west of the Chiang Mai Basin, is a north–south trending active structure which also hosts several hot springs, reflecting ongoing fault movement and hydrothermal discharge (Chansom et al. 2022). Geothermometric data and exploratory drilling throughout the northern region suggest that subsurface reservoir temperatures commonly exceed 150°C, consistent with circulation depths of several kilometres, resulting in the occurrences of several high‐temperature hot springs in the areas (Figure 2a), although some reservoir temperatures could be lower due to dilution by cooler surface waters (Barr et al. 1979; Wood et al. 2018).

In western Thailand, hot springs are situated along the Three Pagodas Fault Zone and the Sri Sawat Fault Zone. The Three Pagodas Fault is a major northwest‐southeast (NW–SE) trending sinistral strike‐slip system extending over 700 km, forming part of a larger fault group, producing hot spring emissions ranging between 40°C and 60°C in western and eastern Thailand (Rattanawong et al. 2020). In southern Thailand, hot springs are situated along the Khlong Marui Fault Zone and Ranong Fault Zone, where fractures in Palaeozoic metamorphic and Mesozoic granitic rocks facilitate upward migration of heated groundwater (Ngansom et al. 2020). Surface temperatures in western and southern springs are generally lower (60°C–70°C) than in northern systems (Figure 2a), reflecting non‐magmatic geothermal gradients and shallower circulation (Ngansom et al. 2023). Additionally, hot springs in southern Thailand are located near coastal zones and often strongly influenced by saline input, resulting in exceptionally elevated sodium and chloride concentrations (Ngansom and Dürrast 2016). Collectively, these fault systems define the principal geothermal pathways in Thailand, where Cenozoic reactivation of inherited basement faults has maintained crustal permeability and enabled convective heat transfer through non‐volcanic crust, creating the widespread distribution of hot springs and associated hydrothermal alteration zones across the country (Ngansom et al. 2020; Ngansom and Dürrast 2021).

According to a classification scheme by Renaut and Jones (2011), the majority of hot springs in Thailand contain bicarbonate‐rich waters (Figure 2b, Table S3), which are formed when CO_2_‐rich fluids condense in shallow groundwater, often where fluids interact with limestone or volcanic rocks. Neutralisation of acidity by rock‐water reactions produces waters dominated by HCO_3_^−^ and Na^+^, with variable sulphate and chloride compositions. Springs are typically clear, with travertine deposits forming where calcium is sufficient and sinter forming where waters are very hot and reach the boiling point (Renaut and Jones 2011). A few hot springs in southern Thailand also exhibited strong saline influence, characterised by exceptionally high sodium (Na^+^) and chloride (Cl^−^) concentrations (Figure 2b, Table S3), indicative of seawater intrusion. This contrasts with bicarbonate‐type waters typical of inland, meteoric, or CO_2_‐rich geothermal systems, where carbon dioxide dissolution and water–rock interaction drive HCO_3_^−^ enrichment (Wei et al. 2021).

According to the DMR of Thailand, all surface hot spring emissions in Thailand are circumneutral or alkaline, with pH ranging approximately from 6.3 to 9.5. True acidic hot springs, with features such as collapses and pits owing to acidic water dissolution (Renaut and Jones 2011), are practically absent. Nationally, hot spring temperature ranges from 32°C to boiling point (DMR). Overall hot spring temperatures (those included in this review) encompassed a range of 32°C–90°C (median = 55.0°C ± 14.0°C) and a pH range of 6.2–10.0 (median = 7.5 ± 1.2). However, the authors would like to note that because around half of the studies included in this review did not provide temperature and/or pH measurements of their studied hot springs, we have compiled additional physicochemical and geochemical data as references (Table S3, Figure 2a). Nevertheless, because these microbiological data were primarily interpreted in association with the original temperatures and pH values taken from those studies, we would continue to use these primary temperature and pH measurements throughout this review, and the reference data were provided only to illustrate the general temperature, pH and geochemical characteristics of overall hot springs in Thailand. In addition, temperature and pH terminologies are defined in Table S4 and are used throughout this review.

## 4 Methodologies of Previous Studies on Hot Spring‐Associated Microorganisms in Thailand

### 4.1 Studied Sites, Research Scopes and Sample Types

Of the 62 hot spring sites included in this review, 24 are located in the northern region, 26 in the southern region, 6 in the western region, 3 in the central region, 2 in the eastern region and 1 in an unspecified region. Hot springs are more common along the north–south direction of Thailand, where numerous faults have formed as a result of extrusion tectonics from the India–Asia continental collision (Singharajwarapan et al. 2012). The most frequently studied sites are also found in the northern region (Figure 2c). San Kamphaeng hot spring was the most investigated, with 14 studies, likely due to its proximity to the city centre and popularity as a tourist attraction. Teppanom hot spring was the second most sampled site (11 studies), followed by Chae Son and Pong Dueat Pa Pae, with 9 studies each (Figure 2c, Table S1).

**FIGURE 3:**
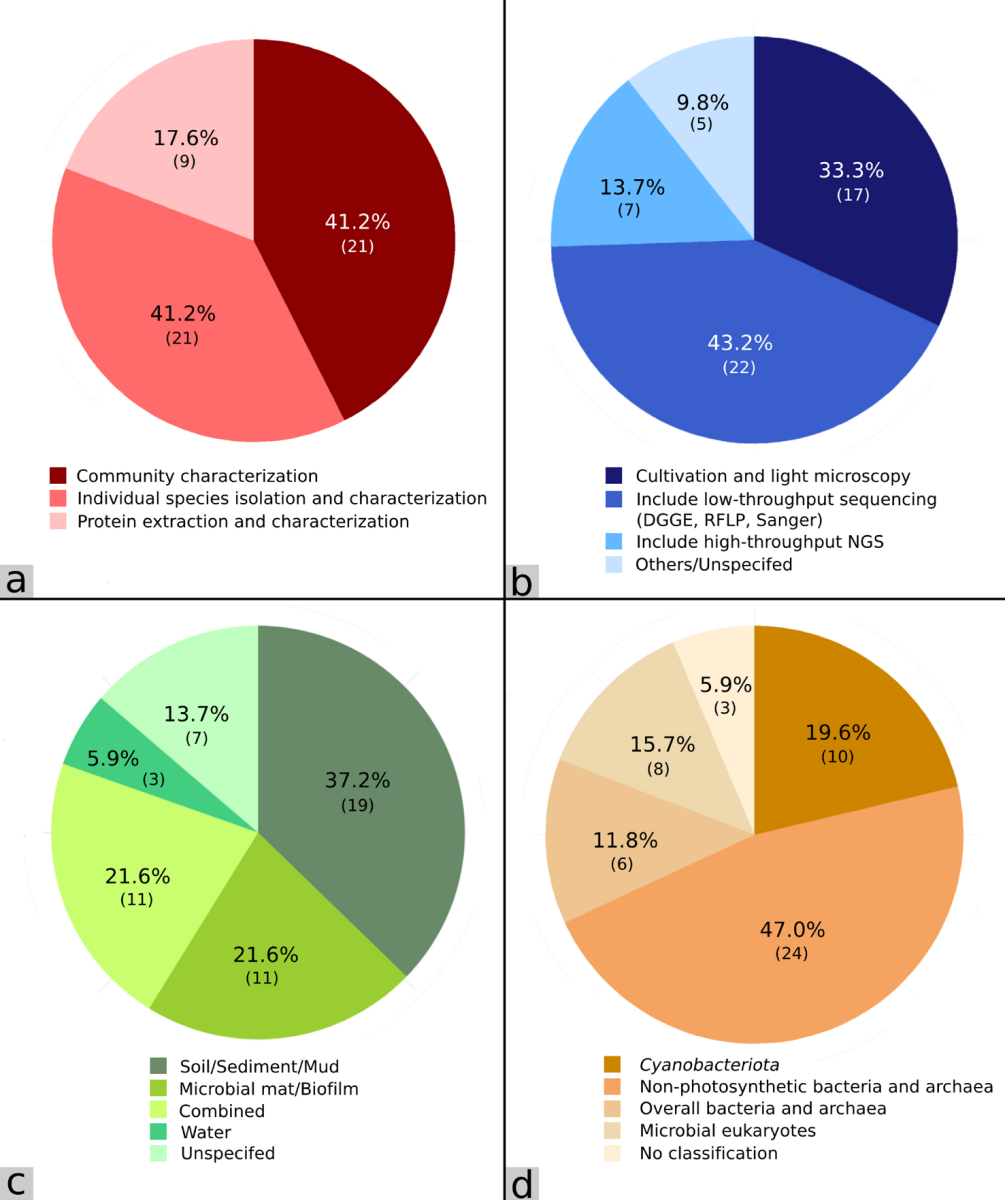
Pie graphs summarising the 51 reviewed studies by (a) research scope, (b) identification approaches employed, (c) collected sample types, and (d) commonly investigated microorganisms/microbial communities.

Out of 51 studies associated with hot spring microorganisms in Thailand, 41.2% characterised hot spring microbial communities and explored their ecology, with another 41.2% focused on the isolation and taxonomic classification of individual species, and 17.6% involved the extraction and characterisation of proteins derived from hot spring microorganisms (Figure 3a, Tables S1 and S5). Accordingly, these microorganisms, including their products, were derived from a variety of hot spring sample types. Soil/sediment/mud was the most studied sample (37.2%), followed by microbial mat/biofilm and combined sample type (21.6% each) (Figure 3c). Water sample was rarely collected as a sole sample type (5.9%) but was rather mixed with others (mostly soil and sediment), potentially due to the need of an extra filtration step to remove unwanted substances (Tables S1 and S5).

### 4.2 Microbial Identification Approaches

One‐third of previous studies (33.3%) were conducted using culture‐dependent methods in which microbial identification was determined solely through light microscopy (Figure 3b, Tables S1 and S5). Despite being highly useful in the identification of microbial morphology, the ability of light microscopy to accurately identify microbial taxonomy is strongly limited owing to a lack of distinguishing morphological features among microorganisms. Comparably, despite cultivation being one of the most robust methods for studying bacterial and archaeal physiological characteristics, metabolic capabilities and optimal physicochemical growth ranges of microorganisms, one of the challenges is that not all microorganisms can be successfully cultivated in the laboratory conditions. It was predicted that more than 80% of microorganisms remain undiscovered/uncultured, especially those from hot spring environments (Ward et al. 1990; Lloyd et al. 2018; Nayfach et al. 2021). Culture‐dependent method is also time‐consuming, limiting the number of microorganisms that can be studied at a time. Nevertheless, given that one‐third of the studies relied solely on morphology‐based identification through light microscopy (Table S1), the authors would like to note that these traditional classifications were retained as originally reported in each study in order to avoid misattributing or altering the identification by the original authors, as we did not have access to their raw samples for verification. However, such classifications should be interpreted with caution as morphological traits may overlap among different taxa, and morphology‐based identifications are generally considered less definitive than those supported by molecular approaches.

In addition, while the traditional approach under the International Code of Nomenclature of Prokaryotes (ICNP) remains the only accepted method for formally classifying newly cultured taxa, the recently developed SeqCode provides a genome‐based framework for naming and classifying uncultured microorganisms, enabling inclusion of a much larger proportion of microbial diversity through a culture‐independent approach (Hedlund et al. 2022). However, SeqCode has not been universally adopted and may not fully reflect the rapidly emerging DNA and metagenomic data. Moreover, SeqCode does not replace the ICNP for the formal description of cultured strains.

Another commonly used method to identify microorganisms associated with hot spring environments in Thailand is the ribosomal RNA gene sequencing technique where analysis of microbial communities is based on 16S or 18S rRNA gene polymerase chain reaction (PCR) amplicon sequences (Figure 3b, Tables S1 and S5). Because the hypervariable regions of the 16S or 18S rRNA genes are highly useful for discriminating microbial taxonomy, taxonomic classification through molecular approaches was the most utilised method among studies on hot spring microorganisms in Thailand. The more conserved regions can help identify high taxonomic ranks and the more rapidly evolving regions can help classify genera or species (Bukin et al. 2019). Almost half of the studies (43.2%) included low‐throughput sequencing techniques in their work (Figure 3b, Tables S1 and S5). Low‐throughput sequencing or traditional molecular techniques such as Polymerase Chain Reaction‐Denaturing Gradient Gel electrophoresis (PCR‐DGGE), PCR‐Restriction Fragment Length Polymorphism (PCR‐RFLP) and Sanger sequencing were commonly applied, and sequencing was generally carried out with only a single DNA fragment per run which was still time‐consuming. In contrast, 13.7% of the studies utilised high‐throughput next‐generation sequencing (NGS) techniques. Owing to its simplicity and cost‐effectiveness, the massively parallel NGS technologies are capable of sequencing millions of fragments, equivalent to hundreds to thousands of genes, simultaneously per one run, and has effectively replaced older microbial identification techniques. Several NGS platforms have been developed to efficiently profile microbial communities, including Roche 454 pyrosequencing (Margulies et al. 2005), Ion Torrent (Rothberg et al. 2011), Illumina (Bennett 2004) and MinION (Jain et al. 2016). Ribosomal RNA gene sequencing has been applied in numerous microbiological studies for microbial profiling from various environments worldwide including hot springs (Woese et al. 1990; Olsen and Woese 1993; Lozupone and Knight 2007; Thompson et al. 2017; Power et al. 2018). Among these NGS studies on Thailand hot springs, two of them also included whole genome sequencing techniques (Tables S1 and S5). Nevertheless, with relatively low numbers of studies that include NGS techniques (13.7%), we propose that more utilisations of modern high‐throughput sequencing technologies may be needed in order to systematically and efficiently capture the snapshots of a wider range of microbial communities associated with hot spring environments, including determining their ecology, biogeography and genomic potentials throughout Thailand.

### 4.3 Commonly Investigated Microbial Taxa

The most reported microbial taxa (47.0%) from Thailand hot springs were of non‐photosynthetic bacterial lineages, particularly Bacilliota and Actinomycetota (Figures 3d and 5, Tables S1 and S5). However, these studies were almost exclusively isolations and characterizations of individual species, rather than characterizations of overall non‐photosynthetic communities (Figure 3d, Tables S1 and S5). On the other hand, 19.6% of studies were devoted solely to Cyanobacteriota, with research ranging from isolation of individual species to community characterisation and extraction of thermostable proteins (Tables S1 and S5). In fact, early studies of hot spring microbial communities in Thailand mostly involved Cyanobacteriota, along with certain green algae, due to the misclassification of Cyanobacteriota as algae. It was not until recently that other bacteria, archaea, including microbial eukaryotes were explored. Indeed, recent studies of overall bacterial and archaeal communities (11.8%) were more common recently because of the arrival of modern molecular techniques which allowed more precise and effective characterisation of various microorganisms. Furthermore, 15.7% of the studies included microbial eukaryotes, although these were mostly limited to only green algae, diatoms and certain members from phylum Amoebozoa.

### 4.4 Community Coverage Assessment

In general, modern well‐designed universal primers cover about 80%–90% of bacterial and archaeal sequences (Mao et al. 2012; Hugerth et al. 2014). However, coverage can vary considerably among primers and datasets. To account for potential methodological biases that could influence the observed distribution of microbial communities, we evaluated the primer set coverage of profiling studies using the SILVA TestPrime tool against the SILVA database SSU‐138.2. Zero mismatches were allowed as coverage proved the highest under strict matching (Mao et al. 2012). When accounted for all 51 studies, the overall estimate of bacterial coverage was 12.00% ± 26.09% and for archaeal coverage, 3.52% ± 14.12% (Table S6). This showed that the existing literature over the past 35 years, at least those included here, likely captured only a little more than one‐tenth of the bacterial community represented in the SILVA database, whereas the archaeal community was alarmingly understudied. These numbers are likely far less than the total natural community, given the high proportion of undiscovered taxa in nature (Ward et al. 1990; Lloyd et al. 2018; Nayfach et al. 2021). Of the 29 studies that employed molecular techniques, only four utilised bacterial primers with coverage above 80%, and only one single study had archaeal primer coverage over 80% (Table S6). In addition, sequencing platform and sequencing depth also substantially affect coverage estimates, as both parameters vary widely among studies and were not often reported, making true coverage calculation impractical. In general, the Illumina platform tends to provide greater coverage and diversity resolution, as well as lower error rates, than older technologies such as 454 pyrosequencing or Ion Torrent, with reported Shannon diversity values around three to four times higher (Claesson et al. 2010; Salipante et al. 2014). Therefore, the distributional patterns reported here should be interpreted as a partial representation of the community composition rather than a comprehensive ecological census of hot spring microorganisms in Thailand.

## 5 Distribution of Photosynthetic Communities in Hot Spring Environments

The studies of microbial communities across hot spring environments in Thailand have profoundly focused on photosynthetic communities. Out of the collective 51 studies on hot spring microorganisms in Thailand, 17 of these (33.3%) included or focused on the ecological distribution of photosynthetic taxa, both prokaryotes and eukaryotes. The most focused photosynthetic group is undoubtedly, Cyanobacteriota, with 10 studies solely dedicated to their distribution, ecology, characterisation and classification (Figure 3d, Table S5). According to the DMR, all hot springs in Thailand are circumneutral or alkaline (pH ~6.3–9.5). This pH range is favourable for the occurrence of Cyanobacteriota, including other photosynthetic prokaryotes and eukaryotes such as Chloroflexota and green algae, since oxygenic photosynthetic activities maximise at neutral to slightly alkaline pH (Dodd and Bidwell 1971; Badger and Andrews 1982). On the other hand, acidic pH may constrain the distribution of bacterial photoautotrophs due to the absence of bicarbonate as a substrate for inorganic carbon photo‐assimilation (Hamilton et al. 2019). In addition, although the presence of acidophilic eukaryotic phototrophs, such as the green algae, *Zygogonium*, and the red algae, Cyanidiophyceae, have been noted from highly acidic hot springs elsewhere (Seckbach 1999; Gross et al. 2001; Schuler et al. 2017), they were absent in the springs included here, where pH remains too high for their growth. This, thus, allows the ubiquitous distributions of mesophilic and alkaliphilic Cyanobacteriota across hot springs in Thailand.

### 5.1 Oxygenic Photosynthetic Bacterial Communities in Hot Springs

**FIGURE 4:**
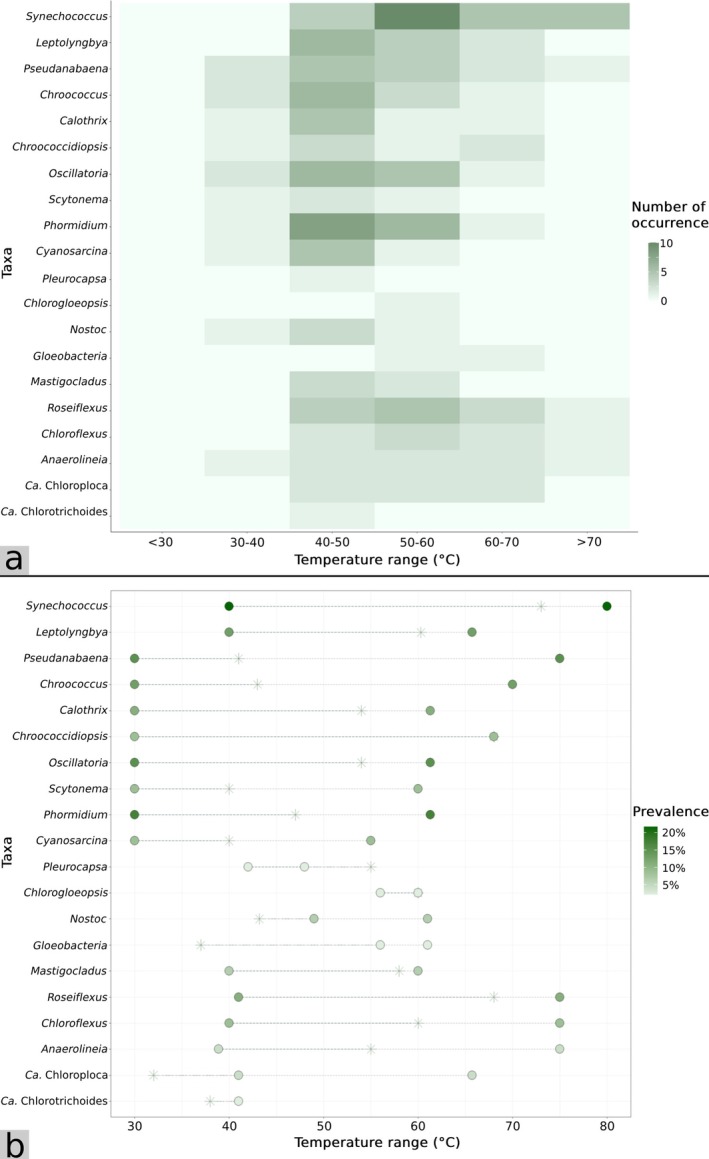
(a) Distribution of photosynthetic bacteria across temperature ranges and the number of occurrence reported across the 51 reviewed studies. Occurrence at each temperature range represents the number of times each taxon was reported within that range across the 51 reviewed studies. A single study could contribute multiple counts for a given taxon if it was reported over multiple temperature intervals or multiple springs. (b) Lower and upper temperature limits (circles) of photosynthetic bacteria from the 51 reviewed studies. Asterisks represent the highest upper temperature limits of the reference taxa when grown under laboratory conditions. Occurrence and prevalence data were compiled from Tables S7 and S8. Reference culturable species are listed in Table S9.

The most detected Cyanobacteriota taxa across hot springs in Thailand were *Synechococcus* (19.61%) and *Phormidium* (15.69%), followed by *Pseudanabaena* and *Oscillatoria* (both at 13.72% each), and *Leptolyngbya* and *Chroococcus* (both at 11.76% each) (Figure 4a,b, Table S7). The distribution pattern of Cyanobacteriota members is noticeable upon temperature variations. The widest distribution ranges across temperatures were up to 45°C (from 30°C to 75°C) which were achieved by members of the *Pseudanabaena* genus and their relatives (Figure 4a,b, Tables S7 and S8). This was followed by *Synechococcus*, which was detected across 40°C temperature ranges (from 40°C to 80°C), *Chroococcus*, which spanned across 40°C temperature ranges (from 30°C to 70°C) and *Chroococcidiopsis*, which was discovered across the 35°C ranges (from 30°C to 65°C). Examples of *Pseudanabaena* members detected from Thailand hot springs included or were closely related to *P. catenate*, *P. galeata* and *Pseudanabaena* sp. PCC 7403 (Sompong et al. 2005, 2008). Several *Synechococcus* species, such as *S. lividus* , *S. bigranulatus*, *S. elongatus* and *Synechococcus* sp. JA‐3‐3Ab were regularly reported (Sompong et al. 2005, 2008; Purcell et al. 2007), along with *Chroococcus thermalis, C.* *globosus* and *C. minutus* (Pitakwapi 1990; Sompong et al. 2005, 2008; Kayan 2014). A few *Chroococcidopsis* species, namely *C. thermalis* and *Chroococcidopsis* sp. A767‐47, the latter a polyextremophilic, ionising‐radiation resistant and desiccation‐tolerant species, isolated from Antarctica dessert (Billi et al. 2000) were also identified (Sompong et al. 2005; Purcell et al. 2007).

Questionably, *Synechococcus* and *Pseudanabaena* were reported from hot springs with temperatures as high as 75°C–80°C (Figure 4a,b, Tables S7 and S8). However, the photosynthetic activity of cyanobacterial species caps with the upper limit of 73°C (Meeks and Castenholz 1971). This seemingly resulted from indirect temperature measurements, in which the recorded temperatures may not represent the actual temperatures where samples were collected but rather adjacent hot spring fluids (Table S1). Indirect temperature measurements were normally employed when direct temperature measurements of collected samples were not practical due to their natures, in which temperature probes cannot be applied to (e.g., the slimy, rubbery and proved resistant textures of the subaerial microbial mat samples or the rocky features of the subaerial deposit samples). However, it has been suggested that the actual temperatures of such subaerial samples may be up to 30°C cooler than the adjacent water (Sriaporn et al. 2024), and thus it is advisable to exercise caution when interpreting these reported temperatures. Additionally, previous studies on the upper temperature limits for growth of *Cyanobacteriota* genera, such as *Pseudanabaena*, from laboratory settings showed as much as 40°C differences from those from hot spring reports (Figure 4b, Table S9). Nevertheless, some of these hot spring microorganisms have not yet been successfully isolated and cultured and might as well represent novel thermophilic strains with elevated thermotolerance.

Not only does the circumneutral‐to‐alkaline pH of the springs support the occurrence of Cyanobacteriota, but the high‐temperature settings of hot springs have also driven their thermal adaptations, with evolved DNA stability and repair mechanisms playing a central role in their ability to persist in such environments (Fagliarone et al. 2017). For example, it was previously reported that the thermotolerance traits of hot spring *Synechococcus* evolved during their diversification in order to occupy higher temperature habitats, resulting in the enhanced thermostability of photosystem II (PSII) and light‐harvesting phycobilisome (PBS), which were acquired through nonsynonymous substitutions in core PSII genes and membrane‐associated genes of PBS (Pittera et al. 2018). This strategy has raised the PSII and PBS inactivation temperatures to be as high as 80°C in some strains, which was higher than the thermal limits for their sustained growth (Pittera et al. 2018). In addition, the higher GC content (61%), compared to its relative marine species (GC = 56.54%) (Zhaxybayeva et al. 2009), may also contribute to the increased thermostability of *Synechococcus* in hot spring environments (Klatt, Inskeep, et al. 2013), since higher thermostability is a well known characteristic of GC‐rich DNA, owing to the three hydrogen bonds binding GC in contrast to the two hydrogen bonds binding AT pairs (Musto et al. 2004; Zheng and Wu 2010). Comparably, in marine *Synechococcus* species, which have a wide latitudinal distribution, ranging from equatorial to polar regions (Zwirglmaier et al. 2008; Pittera et al. 2014), their thermal adaptation mechanisms include the regulation of membrane lipid fluidity by encoding specific fatty acid desaturase enzymes in response to temperature stress to facilitate photosynthetic activities across distinct temperatures (Pittera et al. 2018). Taken together, these adaptive repertoires to thermal stresses reflect the genomic versatility of the *Synechococcus* genus and explain their occurrence across diverse habitats with varying temperatures globally (Zwirglmaier et al. 2008).

The discovery of *Pseudanabaena* members across a strikingly wide temperature range of 45°C (30°C–75°C) from different hot spring studies could potentially be one of the broadest temperature ranges reported from cyanobacterial taxa. Nevertheless, despite being reported from diverse habitats, ranging from freshwater lakes, brackish, epilithic lithophyte, polar regions to geothermal areas, the majority of *Pseudanabaena* members were isolated from cold habitats and most studies were, therefore, rather focused on their cold adaptation (Acinas et al. 2009; Khan et al. 2017; Christodoulou et al. 2023; Aleksovski et al. 2024). *Pseudanabaena* also harbor distinctive musty odour owing to their production of 2‐methylisoborneol (Niiyama et al. 2016; Tuji and Niiyama 2018), in which the synthesis process was suggested to be temperature‐dependent (Park et al. 2024). While their adaptation to high temperatures has not been well‐studied, a previous study established the blooming of *Pseudanabaena* species in microbial community collected from radioactive waste and showed that they had increased polysaccharide production when exposed to high radiation levels (Foster, Boothman, et al. 2020; Foster, Muhamadali, et al. 2020). Produced exopolysaccharides (EPS) surrounding the cells act as a physical barrier against various environmental stresses and, in this case, potentially serve as protective shields to the high heat of hot springs as well (Donot et al. 2012; Nguyen et al. 2020). Similar adaptive mechanisms in upscaling EPS biosynthesis have also been proposed for *Phormidium* as a coping mechanism for thermal stresses (Zampieri et al. 2023).

### 5.2 Anoxygenic Photosynthetic Bacterial Communities in Hot Springs

Along with Cyanobacteriota, another commonly studied photosynthetic bacterial phylum which was abundant and prevalent across various hot springs in Thailand was the filamentous anoxygenic phototrophic Chloroflexota. The most prevalent members included *Roseiflexus* (9.80%), *Chloroflexus* (7.84%) and Anaerolineae (3.92%) (Figure 4a,b). All three taxa shared a similar distribution pattern, spanning across a 35°C temperature gradient (approximately from 40°C to 75°C). Previous literature showed that *Chloroflexus* transcripts were more abundant at 65°C while *Roseiflexus* transcripts were more abundant at 60°C (Bennett et al. 2020). *Roseiflexus* was originally isolated from a hot spring microbial mat in Japan and can grow both photoheterotrophically under anaerobic light conditions and chemoheterotrophically under aerobic dark conditions, and forms distinct rosy, red microbial mats due to their lack of green photosynthetic units such as chlorosomes or chloroplasts (Hanada et al. 2002). These characteristic red mats, co‐occurring with the *Chloroflexus* and cyanobacterial green mats, have been noted from alkaline hot springs in Thailand (Portillo et al. 2009; Sriaporn et al. 2024) as well as elsewhere (Klatt et al. 2011; Bennett et al. 2020).

The prevalent occurrences of *Roseiflexus* across moderate to moderately high temperatures, circumneutral and alkaline hot springs have been widely noted in Thailand hot springs as well as globally (Portillo et al. 2009; Van der Meer et al. 2010; Gaisin et al. 2016; Sriaporn et al. 2024), as their optimal growth occurred at pH of 7.5–8.0 and a temperature range of 45°C–68°C (optimal temperature ~50°C) (Hanada et al. 2002). Although the classification at the species level has not been achieved, most detected *Roseiflexus* in Thailand hot springs were closely related to *R. castenholzii* and *Roseiflexus* sp. RS‐1 (Portillo et al. 2009; Cuecas et al. 2014). Additionally, commonly found *Chloroflexus* species in hot spring environments included *C. aurantiacus* (optimal temperature = 52°C–60°C), *C. aggregans* (optimal temperature = 55°C) and *C. islandicus* (optimal temperature = 55°C) (Pierson and Castenholz 1974; Hanada et al. 1995; Gaisin et al. 2017). However, the species‐level classification was also lacking for *Chloroflexus* from Thai hot springs, with only one study reporting the close phylogenetic relationship between discovered *Chloroflexus* species and *Chloroflexus aurantiacus* Y‐400‐fl, originally isolated from Octopus Spring, YNP (Madigan and Brock 1977). The lack of high‐resolution classification in both Cyanobacteriota and Chloroflexota, including other microbial taxa, emphasises the need for modern high‐throughput sequencing techniques in order to achieve efficient sequencing depth and coverage to resolve fine‐scale taxonomic classification and unveil novel microbial taxa in the hot spring communities of Thailand.

Unlike their ubiquitous Cyanobacteriota neighbours, which have been identified from arrays of habitats, members of the Chloroflexota phylum have been proposed to develop intimate association with hot spring environments. In fact, *Roseiflexus* were found exclusively in geothermal areas associated with the subduction zones of the Circum‐Pacific and Alpine‐Himalayan‐Indonesian orogenic belts as a result of intercontinental prokaryotic taxon divergence (Gaisin et al. 2016). Furthermore, even though the comprehensive study of *Chloroflexus* biogeography has not been established, several literature on *Chloroflexus* has been from hot spring environments (e.g., Pierson and Castenholz 1974; Giovannoni et al. 1987; Weller et al. 1992; Hanada et al. 1995; Nübel et al. 2002; Klatt, Inskeep, et al. 2013; Bennett et al. 2020) and, as far as this review is concerned, the detection of *Chloroflexus* species outside geothermally‐influenced habitats has been unheard of (Nübel et al. 2001).

### 5.3 The Co‐Occurrence of Cyanobacteriota and Chloroflexota and Their Abundance in Hot Spring Environments

The prevalence and abundance of cyanobacterial taxa is a characteristic of circumneutral and alkaline hot springs with moderate to moderately high temperatures, and has been noted globally. Cyanobacteriota genera, such as *Synechococcus*, *Phormidium, Leptolyngbya*, *Pseudanabaena, Calothrix* and *Oscillatoria*, have been identified across wide spectrum of temperatures of Thai hot springs (Sompong et al. 2005; Portillo et al. 2009; Pointing et al. 2024), including elsewhere, for example, YNP (Ward et al. 1998; Klatt et al. 2011; Bennett et al. 2020), Greenland (Roeselers et al. 2007), Iceland (Podar et al. 2020) and New Zealand (Sriaporn et al. 2020). In fact, Cyanobacteriota members, especially *Synechococcus* and *Leptolyngbya* are often reported to co‐exist together with members of Chloroflexota in high abundance, such as those from Mushroom Spring (US) (temperature = 59°C–65°C, pH = 8.5–9.0) (Van der Meer et al. 2010; Becraft et al. 2011; Bennett et al. 2020), Octopus Spring (US) (temperature = 50°C–55°C, pH = 8.2) (Allewalt et al. 2006; Van der Meer et al. 2010; Bennett et al. 2020), Whangapaoa (New Zealand) (temperature = 48.1°C, pH = 8.5) (Sriaporn et al. 2020), including many alkaline springs in Thailand (e.g., from Pong Dueat Pa Pae, Chae Son, Boekleung and Mae Fang hot springs) (Portillo et al. 2009; Cuecas et al. 2014; Pointing et al. 2024; Sriaporn et al. 2024, 2025). Both of these photosynthetic phyla occupied around or even more than half of the microbial mat community compositions in certain hot spring settings (e.g., Klatt et al. 2011; Sriaporn et al. 2023). Previous studies have demonstrated an elevated growth rate of *Chloroflexus* spp., when grown in a dual culture with *Synechococcus* spp. (Castenholz and Pierson 1981). It has been proposed that the filamentous anoxygenic phototroph *Chloroflexus* relies on small organic carbon compounds produced by Cyanobacteriota, in which they utilise for their fermentation and photoheterotrophic growth (Van der Meer et al. 2010; Klatt, Inskeep, et al. 2013), resulting in their spatial co‐occurrence in several circumneutral and alkaline geothermal environments.

In addition, photoautotrophic Cyanobacteriota are among the major primary producers in alkaline hot springs between 60°C and 72°C, whereas phototrophic Chloroflexota mainly contribute to carbon fixation in hot springs (Bennett et al. 2020). Because of their photosynthetic natures, these two taxa are found residing primarily in the top layers of microbial mats, where they are able to efficiently receive energy from the sun in order to perform light‐induced photochemical reactions. Deeper mat layers (e.g., 20–30 cm from the surface), where sunlight cannot penetrate and oxygen concentration is depleted, are inhabited by a very small number of Cyanobacteriota and Chloroflexota (Kanokratana et al. 2004). On the contrary, these lower mat layers are resided in by heterotrophic microorganisms which rely on organic matters produced by Cyanobacteriota and Chloroflexota (Giovannoni et al. 1987; Ward et al. 1998; Klatt et al. 2011; Klatt, Liu, et al. 2013). This colony partitioning is a common feature and has been observed in various alkaline hot springs in Thailand and elsewhere (Portillo et al. 2009; Bennett et al. 2020; Sriaporn et al. 2025).

### 5.4 Photosynthetic Microbial Eukaryotes in Hot Spring Environments

Apart from photosynthetic bacterial taxa, photosynthetic microbial eukaryotes were also reported from circumneutral‐to‐alkaline, moderate temperature hot springs across Thailand. In fact, it has been shown that the biomass of prokaryotes and eukaryotes (estimated from 16S and 18S rRNA gene copies per gramme of sample) in circumneutral and alkaline hot springs was almost similar (Sriaporn et al. 2020). Yet, studies on the ecological distribution and characterisation of microbial eukaryotes from hot springs in Thailand remain scarce and are not well‐documented. Here we noted major eukaryotic photosynthetic taxa that have been isolated from Thailand hot spring environments, the green algae, namely Chlorophyta and Charophyta and diatoms (Duangjan et al. 2017; Pruetiworanan et al. 2018; Pumas et al. 2018), along with a few identifications of the golden algae Chrysophyceae, the plastid‐containing Cryptophyceae, Euglenophyta and a SAR clade member Dinoflaggellates (formerly described as Pyrrophyta) (Pitakwapi 1990; Sompong 2001). Other identified green algae, classified through light microscopy, included *Acutodesmus Obliquus*, *Chlamydomonas parallestriata*, *Cosmarium lundellii* , *Selenastrum bibraianum* , *Desmodesmus communis* and *Staurastrum muticum* (Duangjan et al. 2017). In addition, more than 40 species of diatoms have been identified from eight hot springs with a temperature range of 38.4°C–85.0°C and a pH range of 6.8–8.8 (Pumas et al. 2018). Of these, *Diatomella balfouriana* , *Achnanthidium exiguum* and *Anomoeoneis sphaerophora* were the most abundant diatom species. To date, the classification and characterisation of hot spring eukaryotic communities in Thailand have been achieved mainly by light microscopy. This, thus, highlights the need for modern high‐throughput sequencing techniques in order to produce an accurate, systematic and comprehensive collection of eukaryotic communities associated with geothermal environments in Thailand.

## 6 Distribution of Non‐Photosynthetic Communities in Hot Spring Environments

Out of 51 studies, 24 of them specifically addressed non‐photosynthetic microorganisms found across hot springs in Thailand (Figure 3d, Table S5). However, 18 of these 24 studies focused only on the isolation and characterisation of individual species. Moreover, another three studies did not provide any taxonomic classifications, with one reporting just the numbers of culturable colonies, and the other two focusing on the extraction of thermostable enzymes derived from hot spring bacteria (Tang et al. 2006; Techaoei et al. 2007; Tirawongsaroj et al. 2008). Therefore, we gathered there were only six studies dedicating to the characterisation of non‐photosynthetic hot spring microbial communities in Thailand (Kanokratana et al. 2004; Purcell et al. 2007; Hniman et al. 2011; Tingthong and Ganso 2012; Saimmai et al. 2017; Bumrungthai et al. 2020), with another five studies that included both photosynthetic and non‐photosynthetic hot spring microbial communities (Portillo et al. 2009; Cuecas et al. 2014; Pointing et al. 2024; Sriaporn et al. 2024, 2025) through a combination of molecular techniques such as DGGE, RFLP and NGS, coupled with culture‐dependent methods and light microscopy (Table S5).

**FIGURE 5:**
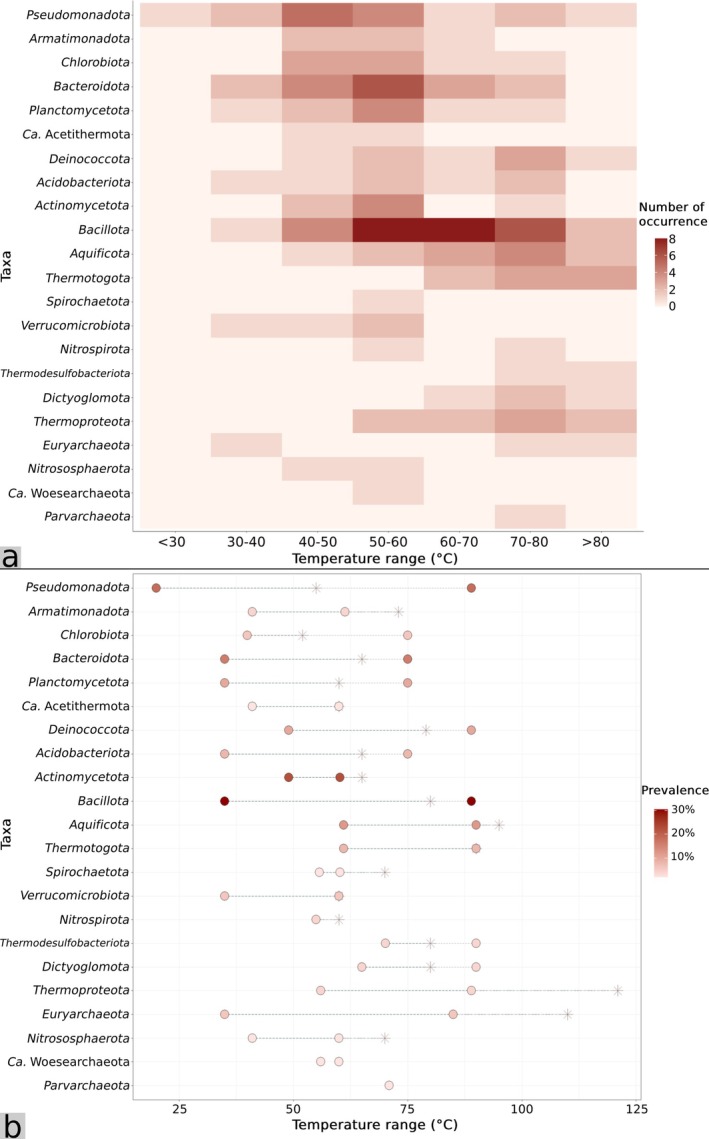
(a) Distribution of non‐photosynthetic bacteria and archaea across temperature ranges and the number of occurrence reported across the 51 reviewed studies. Occurrence at each temperature range represents the number of times each taxon was reported within that range across the 51 reviewed studies. A single study could contribute multiple counts for a given taxon if it was reported over multiple temperature intervals or multiple springs. (b) Lower and upper temperature limits (circles) of non‐photosynthetic bacteria and archaea from the 51 reviewed studies. Asterisks represent the highest upper temperature limits of the reference taxa when grown under laboratory conditions. Occurrence and prevalence data were compiled from Tables S7 and S8. Reference culturable species are listed in Table S9. Note that Ca. Woesearchaeota and Parvarchaeota currently do not have any successfully isolated and culturable members.

In general, phylogenetically diverse taxa of non‐photosynthetic microorganisms have been studied and reported, with more than 20 phyla of bacteria and archaea recognised across geothermal areas in Thailand (Figure 5a,b). These non‐photosynthetic bacteria and archaea were identified at temperatures exceeding those inhabited by photosynthetic taxa. Non‐photosynthetic microorganisms harbour more diverse functional capabilities and are able to utilise variety of hot spring minerals as energy and electron sources (i.e., chemotrophs). Examples of chemoautotrophic inhabitants of geothermal areas are ammonia oxidisers (e.g., *Nitrospira* and *Nitrososphaerota*), sulphur oxidisers (e.g., *Acidithiobacillus* which also can oxidise thiosulfate, sulphide and sulphite) and hydrogen producers (e.g., *Bacillus* and *Venevibrio*) which are found to be common residents among the non‐photosynthetic communities of hot springs in Thailand and elsewhere (Hniman et al. 2011; Sriaporn et al. 2021; Zhang et al. 2023). Other non‐photosynthetic microorganisms are heterotrophs consisting of various bacterial taxa, including microbial eukaryotes, and have also been identified from hot spring environments globally (Krebs et al. 2014; Colman et al. 2016; Oliverio et al. 2018; Power et al. 2018; Sriaporn et al. 2020). Overall, all of these non‐photosynthetic taxa can be found across geothermal areas globally including hot springs in Thailand.

### 6.1 Non‐Photosynthetic Bacterial Communities in Hot Spring Environments

The most studied non‐photosynthetic bacteria were of the phyla Bacillota (29.41%) and Actinomycetota (21.57%), in which several studies focused solely on individual species isolation and characterisation (Figure 5a,b, Tables S7 and S8). For non‐photosynthetic bacterial community characterisation studies, phyla commonly found in the communities across hot springs in Thailand were Pseudomonadota and Bacteroidota (with 17.65% and 15.69% prevalence, respectively), followed by Aquificota (11.76% prevalence), Planctomycetota (9.80%) and Deinococcota (9.80%) (Figure 5a,b, Tables S7 and S8). Of these taxa, Pseudomonadota was recorded to harbour the widest distribution range across temperature gradients, encompassing almost 70°C (20°C–89°C). Identified Pseudomonadota members included Alphaproteobacteria and Betaproteobacteria (Purcell et al. 2007). However, higher classification resolution has not been achieved. Accordingly, members belonging to Bacteroidota detected across hot spring sites included those related to *Bacteroides acidofaciens* and *Flavobacterium* species. Some of these identified sequences were also phylogenetically related to species reported from other hot spring sites globally such as YNP and Japan (Hirayama et al. 2005; Portillo et al. 2009). Nevertheless, phylogenetic analysis revealed that the majority of identified bacteria in Thailand hot springs had < 96.5% 16S rRNA sequence similarity to publicly available species, implying that a large fraction of bacterial community from hot springs in Thailand remained uncultured and unclassified (Kanokratana et al. 2004; Portillo et al. 2009). Moreover, although the global bacterial databases have been expanding considerably, the focus on phylogenetics from recent hot spring microbiology studies in Thailand has generally been somewhat lacking, and thus most phylogenetic relationships to established global taxa remain unclear. In addition, like the case with photosynthetic bacteria, we noticed that certain studies claimed the upper temperature limits of several hot spring microorganisms to be higher than their optimal growth ranges when grown in a culture in laboratory settings (Figures 4b and 5b, Table S9). Overall, we observed from these studies that across hot springs in Thailand, temperatures influence bacterial community composition and diversity (Figures 4a and 5a), given the limited range of pH observed here. However, in other geothermal regions with broader pH ranges, both temperature and pH are commonly reported as major drivers of hot spring microbial community composition and diversity (Miller et al. 2009; Krebs et al. 2014; Sharp et al. 2014; Colman et al. 2016; Power et al. 2018).

Thermophilic bacteria acquire several thermal adaptation strategies in order to cope with increased temperatures. For certain bacterial lineages, such as *Acidithiobacillus* (of Pseudomonadota), it was shown that they have reduced genome sizes, higher GC and proline‐encoding contents, and streamlined genomes as mechanisms to tackle the extremely high temperatures of hot springs (> 90°C) (Sriaporn et al. 2021). Other bacteria reported from extremely high‐temperature springs (> 90°C), such as Thermotogota, possess thermotolerant structures like toga sheath which envelopes the outer membrane (Huber et al. 1986). Aquificota, with members such as *Aquifex* and *Venenivibrio*, have frequently been described from several high‐temperature hot springs as well (Reysenbach et al. 2000; Power et al. 2024), although it is still being debated whether they have acquired numerous thermotolerant genes via lateral gene transfer, which likely aid their adaptation to high‐temperature environments, or they were originally of thermophilic origin (Eveleigh et al. 2013; Giovannelli et al. 2017; Leng et al. 2023).

### 6.2 Archaeal Communities in Hot Spring Environments

The first discovery of archaea was from geothermal environments, and since then, a great variety of archaea has been discovered from hot springs worldwide (Brock 1967; Brock and Freeze 1969; Brock et al. 1972). Previous literature noted the correlations between archaeal relative abundance and diversity and increasing temperatures, reflecting their preferences for high‐temperature niches (Sriaporn et al. 2020; Karaseva et al. 2024; Qi et al. 2024) and the early characterisation of archaea solely as extremophiles (Woese et al. 1978). Archaeal colonisation in high temperatures was achieved owing to tetraether lipids in their cytoplasmic membranes which allow them to maintain membrane integrity at high‐temperature environments (Woese et al. 1978; Albers et al. 2000; Konings et al. 2002).

Despite their intimate relationships with high temperatures, existing studies involving archaeal communities from hot springs in Thailand are considered scarce, with only five studies including the characterisation of archaeal communities (Table S5). Moreover, these studies often showed archaeal communities as drastically less abundant than bacterial, with an average of < 1% relative abundance in the hot spring communities (e.g., Bumrungthai et al. 2020; Sriaporn et al. 2024, 2025). Among described archaeal taxa, Euryarchaeota was the most prevalent phylum (with 5.88% prevalence), spanning a 50°C temperature range (35°C–85°C), followed by Thermoproteota and Dictyoglomota (each with 3.92% prevalence), encompassing a 30°C temperature range (55°C–85°C) and 25°C temperature range (65°C–90°C), respectively (Figure 5a,b, Tables S7 and S8). Other minor archaeal phyla included Nitrososphaerota, Woesearchaeota and Parvarchaeota, in which each was mentioned only once among these studies. In general, the archaeal community abundance in moderate‐to‐high temperature, circumneutral‐to‐alkaline hot springs is comparably lower than those from highly acidic hot springs where archaea tend to dominate and equal bacterial abundance (Colman et al. 2018; Sriaporn et al. 2023; Qi et al. 2024) owing to their (hyper)extremophilic natures (Brock 1967). Moreover, the occurrences of certain archaeal linages, such as *Sulfolobus* and *Thermoplasma*, are limited mainly to highly acidic habitats (with high abundance at pH ~ 2.0–3.0) or extremely high temperatures rather than circumneutral and moderate hot spring environments (Karaseva et al. 2024; Qi et al. 2024). Nevertheless, it is worth noting that the underestimation of archaeal lineages among Thai hot springs also likely results from methodological limitations, such as the uses of V4 primers (e.g., 341F/805R) which work against archaeal detection (Walters et al. 2016), or the uses of universal primers specifically designed for bacteria to target archaeal sequences (Frank et al. 2008) (Table S1). Moreover, the extremely low archaeal primer coverage (3.52% ± 14.12%) certainly contributed to the minor archaeal fraction across Thai hot springs (Table S6). Therefore, we recommend including archaeal‐inclusive amplicons or shotgun metagenomes for future survey of hot spring archaeal communities across Thailand in order to improve community recovery.

### 6.3 Non‐Photosynthetic Microbial Eukaryotic Communities in Hot Spring Environments

As far as this review is concerned, there is no study on the characterisation of non‐photosynthetic microbial eukaryotic community from Thai hot springs. However, two studies reported the presence/absence of pathogenic free‐living amoeba, *Naegleria* and *Acanthamoeba*, from several hot springs across Thailand (Lekkla et al. 2005; Nunthawarasilp et al. 2007). In addition, a few studies involving isolation and characterisation of novel thermostable enzymes from a fungal species from Boekleung hot spring (Tang et al. 2006, 2008) suggested the presence of fungal communities in Thailand hot spring environments. In fact, previous studies based on NGS have shown the presence of diverse phylogenetic clades of fungi and other eukaryotes in hot springs elsewhere globally, such as Alveolata, Amoebozoa, Archaeplastida, Rhizaria, Stamenopiles, Opisthokonta and Excavata (Oliverio et al. 2018; Sriaporn et al. 2020). This, therefore, suggests the unexplored territory of hot spring microbial eukaryotic communities across Thailand that may harbour many potentials for biochemical applications in addition to those from bacterial and archaeal species.

## 7 Summary, Limitations and Suggestions

Geothermal ecosystems are hotspots for unique and diverse microbial populations. Here we summarise and review the ecological distribution of the bacterial, archaeal and microbial eukaryotic communities from 51 studies, covering 62 hot springs, spanning across 21 provinces along the northern, central, eastern, western and southern regions of Thailand. Previous work encompassed various research scopes, and microbial characterisation and classification were conducted through both traditional and modern techniques. Commonly characterised microbial communities were of Cyanobacteriota lineages, in which prevalently found genera included *Synechococcus* and *Phormidium*. While temperature is one of the main influencers of hot spring microbial community compositions in Thailand, the occurrence of certain cyanobacterial members, for example, *Pseudanabaena*, *Synechococcus*, *Chroococcus* and *Chroococcidiopsis* were reported at wide‐ranging temperature ranges of 30°C–75°C, 40°C–80°C, 30°C–70°C and 30°C–65°C, respectively. Spatial occupancy between Cyanobacteriota and anoxygenic photosynthetic bacteria, *Chloroflexus* and *Roseiflexus*, were often noted in alkaline springs. For non‐photosynthetic hot spring bacteria, Pseudomonadota and Bacteroidota were the majority of the communities, while members from Bacillota were the most isolated. Pseudomonadota also harbour the widest distribution range across temperature gradients, encompassing almost 70°C spectrum (20°C–89°C). Archaea were almost absent and rarely noted in the community, possibly due to methodological limitations. Microbial eukaryotes, such as green algae, diatoms and *Amoebozoa*, were isolated and classified but their communities have not yet been fully characterised.

Common caveats identified among existing literature include the impractical measurements of hot spring temperatures. Temperature measurements were often taken solely from hot spring water, yet subaerial samples, such as sediments or biofilm on rock surfaces, could potentially be as much as 30°C cooler than their adjacent water due to exposure to cooler air temperatures. Moreover, certain samplings took place across multiple spatial differences, but only a single temperature measurement was recorded. Overall, these caveats present limitations to the available data and an extent of caution is needed when interpreting the reported temperature limits of each microorganism. On the other hand, hot spring systems are dynamic with multiple stressors (e.g., heat, UV radiation and wet–dry cycles) that may potentially cause adaptation and evolution of microorganisms therein, and the occurrence of novel thermophilic strains cannot be entirely excluded.

In addition, we suggest that future classification and characterisation work should include more modern high‐throughput molecular approaches such as NGS techniques, with well suited primer sets, in order to systematically and efficiently detect a diverse array of bacterial, archaeal and microbial eukaryotic communities from hot spring environments across Thailand, including achieving fine‐scale taxonomic classification. The generation of large‐scale microbial profile data which can potentially be integrated into national hot spring‐related databases will likely be significant and useful for future biodiscovery and applications in various fields, including serving as a means to assess the conservation, recreational and resource development value of the microbial component of geothermal ecosystems.

## Author Contributions

Conceptualisation: C. Sriaporn and J. Pekkoh. Resources: K. Phinyo and J. Pekkoh. Writing – original draft preparation: C. Sriaporn. Writing – review and editing: C. Sriaporn, K. Phinyo, U. Sompong, J. Homnan and J. Pekkoh. Visualisation: C. Sriaporn, J. Homnan and J. Pekkoh. Supervision: S. Komonjinda and J. Pekkoh. Funding acquisition: S. Komonjinda and J. Pekkoh.

## Funding

This work was supported by Chiang Mai University.

## Conflicts of Interest

The authors declare no conflicts of interest.

## Supporting information


**Figure S1:** The 16 active fault lines across Thailand. The base map was derived from Esri, USGS and NOAA. Fault line data were retrieved and modified from the Department of Mineral Resources of Thailand (https://library.dmr.go.th/elib/cgi‐bin/opacexe.exe?op=mmvw&db=Main&skin=s&mmid=11175&bid=36776).

**Table S1:** Metadata of the 51 studies on hot spring‐associated microorganisms in Thailand included in this review.
**Table S2:** Keywords used for literature search. Keywords were applied both individually and in combinations to refine search results.
**Table S3:** Reference temperature, pH and geochemical characteristics of hot springs in Thailand compiled from additional sources.
**Table S4:** List of terminologies and their definitions used in this review.
**Table S5:** Summary of reviewed studies based on study characteristics.
**Table S6:** Primers coverage as calculated by SILVA TestPrime tool against SILVA database SSU‐138.2 with RefNR collection and 0 mismatches.
**Table S7:** Distribution and frequency of photosynthetic and non‐photosynthetic bacteria and archaea across temperature ranges in the 51 reviewed studies. Occurrence at each temperature range represents the number of times each taxon was reported within that range across the 51 reviewed studies. A single study could contribute multiple counts for a given taxon if it was reported over multiple temperature intervals.
**Table S8:** Lower and upper temperature limits of photosynthetic and non‐photosynthetic bacteria and archaea, and the number of studies reporting each taxon across the 51 reviewed studies. Each taxon was counted once per study, regardless of the number of occurrences reported within that study.
**Table S9:** Reference isolated and culturable species under laboratory conditions in which the highest temperature limits of the taxa were identified.

## Data Availability

The data that supports the findings of this study are available in the Supporting Information of this article.
