# Supplementary material for: Microbial Distributions Across Wide‐Ranging Temperature Gradients of Hot Springs in Thailand: A Review of 35 Years of Research on Hot Spring‐Associated Microorganisms
Source: Environ Microbiol Rep. 2026 Feb 18;18(1):e70302. doi: 10.1111/1758-2229.70302 (PMC12916155; doi:10.1111/1758-2229.70302)
Supplement: Supplementary file 1 — Figure S1: The 16 active fault lines across Thailand. The base map was derived from Esri, USGS and NOAA. Fault line data were retrieved and modified from the Department of Mineral Resources of Thailand (https://library.dmr.go.th/elib/cgi‐bin/opacexe.exe?op=mmvw&db=Main&skin=s&mmid=11175&bid=36776). [file EMI4-18-e70302-s001.docx]

**Supplementary Figure**


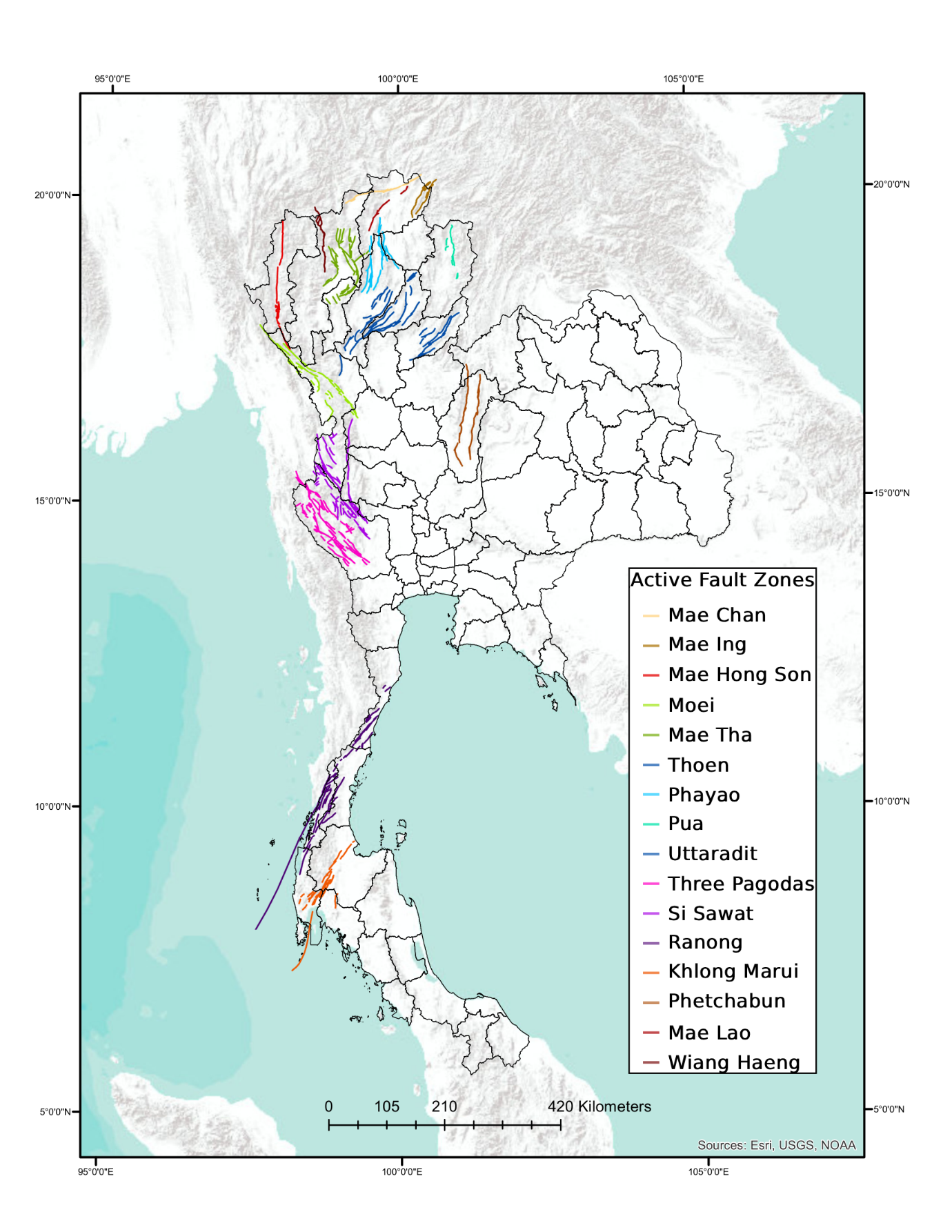


Supplementary Figure 1. The 16 active fault lines across Thailand. The base map was derived from Esri, USGS, and NOAA. Fault line data were retrieved and modified from the Department of Mineral Resources of Thailand (https://library.dmr.go.th/elib/cgi-bin/opacexe.exe?op=mmvw&db=Main&skin=s&mmid=11175&bid=36776).
